# Functional alignment in robotic total knee arthroplasty provides favourable outcomes and minimal early revisions: A systematic review and meta‐analysis

**DOI:** 10.1002/ksa.70226

**Published:** 2025-12-08

**Authors:** Vasileios Giovanoulis, Angelo V. Vasiliadis, Luca Andriollo, Pietro Gregori, Konstantinos Dretakis, Christos Koutserimpas, Sébastien Lustig

**Affiliations:** ^1^ Department of Orthopaedics Surgery and Sports Medicine Hôpital de la Croix‐Rousse Lyon France; ^2^ Department of Anatomy University of Thessaly Volos Greece; ^3^ Ortopedia e Traumatologia, Fondazione Poliambulanza Istituto Ospedaliero Brescia Italy; ^4^ Fondazione Policlinico Universitario Campus Bio‐Medico Roma Italy; ^5^ 2nd Department of Orthopaedics Hygeia General Hospital of Athens Athens Greece; ^6^ School of Rehabilitation Health Sciences University of Patras Patras Greece; ^7^ IFSTTAR, LBMC UMR_T9406, Univ Lyon, Claude Bernard Lyon 1 University Lyon France

**Keywords:** functional alignment, functional knee positioning, personalised alignment, robotic knee, robotic TKA

## Abstract

**Purpose:**

Functional alignment (FA) has emerged as a personalised alignment strategy in total knee arthroplasty (TKA), enabled by robotic‐assisted technology. Although early clinical results are encouraging, evidence remains heterogeneous and long‐term safety is uncertain. This systematic review aimed to evaluate functional outcomes, and early revision risk of robotic‐assisted TKA performed under FA principles with a minimum follow‐up of 2 years.

**Methods:**

This review was conducted according to PRISMA guidelines and registered in PROSPERO (CRD420251134340). PubMed/MEDLINE and Scopus were searched up to August 2025. Eligible studies included adult patients undergoing robotic‐assisted primary TKA with FA, reporting outcomes at ≥2 years' follow‐up. Data on demographics, robotic system, implant type, patient‐reported outcome measures (PROMs), range of motion (ROM), complications, and revision rates were extracted. Risk of bias was assessed using ROB 2 and ROBINS‐I. A random‐effects single‐arm meta‐analysis of pooled proportions was performed for early aseptic revision risk.

**Results:**

Twenty studies (5155 TKAs) were included, with a weighted mean follow‐up of 2.5 years. All procedures were performed using the image‐based robotic system. Postoperative PROMs were consistently high: Knee Society Score (KSS) Knee 92.4–94.5, KSS Function 91.4–93.4, and FJS 74.8–77.5. Kujala scores indicated minimal anterior knee pain (88.1–93.2). ROM improved from a mean preoperative flexion of 119.3° to 123°–124.8°. Complications were infrequent, most commonly stiffness requiring manipulation under anaesthesia. Across all cohorts, 18 aseptic revisions were reported (pooled proportion 0.35%, 95% CI 0.23–0.55). The single‐arm meta‐analysis of pooled proportions showed a negligible pooled early revision risk (rounded to 0.00; 95% CI 0.00–0.01; *I*² = 0%). Outcomes were comparable across age, gender, fixation type, and implant constraint, although obese patients accounted for all mechanical failures.

**Conclusion:**

Robotic‐assisted TKA performed under FA principles yields favourable short‐ to mid‐term functional outcomes and a low rate of early aseptic revision.

**Level of Evidence:**

Level III, systematic review and meta‐analysis.

**Abbreviations**
AKPS: Anterior Knee Pain Scale
CI: confidence interval
EQ‐5D: EuroQol‐5D
FA: functional alignment
FJS: Forgotten Joint Score
KA: kinematic alignment
KOOS: Knee injury and Osteoarthritis Outcome Score
KSS: Knee Society Score
OKS: Oxford Knee Score
PRISMA: Preferred Reporting Items for Systematic Reviews and Meta‐Analyses
PROMS: patient‐reported outcome measures
ROM: range of motion
TKA: total knee arthroplasty
WOMAC: Western Ontario and McMaster Universities Osteoarthritis Index

## INTRODUCTION

Functional alignment (FA), also referred to as functional knee positioning, has emerged as a personalised alignment philosophy in total knee arthroplasty (TKA) that aims to restore native knee biomechanics by balancing bony anatomy and soft tissue tension in three dimensions [5, 22]. Unlike mechanical alignment, which imposes a uniform neutral axis, FA individualises implant positioning based on each patient's limb morphology, ligamentous laxities, and anterior compartment behaviour [16, 39]. This approach relies on the precise intraoperative evaluation of mediolateral gaps in extension and flexion, joint line preservation, and component orientation tailored to the patient's phenotype. Such detailed assessment and execution are only feasible with robotic‐assisted platforms, which provide accurate, real‐time data on implant positioning, gap balancing, and kinematic behaviour [5, 16, 22, 39].

This newly introduced philosophy of alignment has demonstrated promising early outcomes, but it remains under active investigation [22, 33, 38]. FA is intrinsically linked to robotic platforms, which have been shown to be safe and accurate, providing three‐dimensional implant planning together with precise evaluation of mediolateral gaps in both extension and flexion [13, 14, 24]. While FA has been associated with favourable kinematics, reduced need for soft tissue releases, and high levels of patient‐reported satisfaction, the available evidence is still relatively limited and heterogeneous, consisting mainly of single‐center cohort studies [42, 44]. Important questions remain regarding its long‐term safety, reproducibility, and potential impact on revision risk when compared with traditional alignment strategies. Given the growing adoption of robotic systems worldwide, there is a pressing need to synthesise the existing evidence to clarify the clinical performance of FA in robotic TKA.

The objective of this systematic review and single‐arm meta‐analysis was to critically evaluate the outcomes of robotic‐assisted TKA performed under FA principles, with a specific focus on patient‐reported outcomes, complication rates, and early revision risk at a minimum of 2 years' follow‐up. We hypothesised that robotic‐assisted FA would provide excellent functional results and implant survivorship without increasing the incidence of complications or early revisions.

## METHODS

This systematic review was conducted in accordance with the Preferred Reporting Items for Systematic Reviews and Meta‐Analyses (PRISMA) guidelines [36]. The protocol was prospectively registered with the International Prospective Register of Systematic Reviews (PROSPERO) (registration number: CRD420251134340).

### Search strategy

A comprehensive search of PubMed/MEDLINE and Scopus databases was performed from database inception to August 2025. The following search string was applied: (“total knee arthroplasty” OR “TKA” OR “total knee replacement” OR “TKR”) AND (“functional alignment” OR “functional knee position” OR “FA” OR “FKP”) AND (robotic OR “robotic‐assisted” OR “robot‐assisted”).

The search was restricted to studies published in English. Reference lists of included full‐text articles were screened manually to identify additional eligible studies.

### Eligibility criteria

Studies were considered eligible if they included adult patients undergoing primary robotic‐assisted TKA under FA principles, and if they reported outcomes at a minimum follow‐up of 2 years. Acceptable study designs were randomised controlled trials, prospective or retrospective comparative cohort studies, or large case series with at least 50 patients. To be included, studies also needed to report at least one validated patient‐reported outcome measure, such as the Knee Society Score (KSS), the Forgotten Joint Score (FJS), the Western Ontario and McMaster Universities Osteoarthritis Index (WOMAC), the Oxford Knee Score (OKS), the Kujala score, the Knee injury and Osteoarthritis Outcome Score (KOOS), or the EuroQol‐5D (EQ‐5D). Studies were excluded if they involved revision or partial knee arthroplasty, small case series with fewer than 50 patients, cadaveric or biomechanical investigations, expert opinions, narrative reviews, or non‐English language publications.

### Study selection and data extraction

Study selection was carried out independently by two reviewers (V.G., AV.V.), who first screened titles and abstracts, and subsequently reviewed the full texts of potentially eligible articles. Discrepancies were resolved by discussion with a third reviewer (C.K). Data extraction was also conducted independently and included study design and information, patient demographics, robotic system used, and comparator groups where applicable [2, 6, 12, 13]. Outcomes extracted included patient‐reported outcome measures, range of motion, complications, as well as revision rates. The indications for the revisions were also recorded.

### FA strategy

Across the included literature, most cohorts applied FA strategy within a standardised set of planning boundaries. Fourteen of the twenty studies (Andriollo et al. [1, 2, 3], Diquattro et al. [11, 12], and Koutserimpas et al. [20, 23, 25, 27, 28, 29, 30] explicitly reported using the image‐based robotic workflow described by Shatrov et al., which targets HKA 174°–180°, femoral positioning from 3° varus to 6° valgus, tibial positioning at 0°–6° varus, femoral rotation 0° internal rotation–6° external rotation [39]. The remaining cohorts applied comparable limits: Nixon et al. [37], Young et al. [44], Yu et al. [45] and Manara et al. [34] followed similar FA principles and implant positioning ranges. Choi et al. [6] positioned both components within ±3° in the coronal plane, with an overall limb alignment of 0–3°. Clark et al. [8] allowed tibial positioning up to 6° varus and femoral positioning up to 6° valgus, while Daffara et al. [10] reported tibial alignment within 4° varus and 2° valgus. Yang et al. [43] targeted component positioning within ±5° of varus/valgus.

### Statistical analyses

Continuous variables were extracted as means and standard deviations, while categorical variables were recorded as frequencies or percentages. A single‐arm meta‐analysis of pooled proportions was performed to calculate the incidence of aseptic revision among FA cohorts. Pooled event rates with 95% confidence intervals were generated using a random‐effects model.

Data synthesis and meta‐analyses were performed using Review Manager (RevMan, version 5.4; Cochrane Collaboration) and R software (version 4.3.2) with the ‘meta’ and ‘metafor’ packages. Heterogeneity was assessed with Cochran's Q, τ2, and *I* ^2^ (with ~25%, ~50%, ~75% interpreted as low, moderate, high). Authors also reported a 95% prediction interval when estimable. Small‐study effects/publication bias were assessed with Peters' test for the proportion meta‐analysis and Egger's regression for the incidence‐rate meta‐analysis (two‐sided *α* = 0.05).

### Quality assessment

The risk of bias was assessed using the RoB 2 tool for randomised controlled trials and the ROBINS‐I tool for non‐randomised studies. The certainty of evidence was rated as very low, low, moderate, or high based on the domains of risk of bias, inconsistency, indirectness, imprecision, and publication bias.

## RESULTS

**Figure 1:**
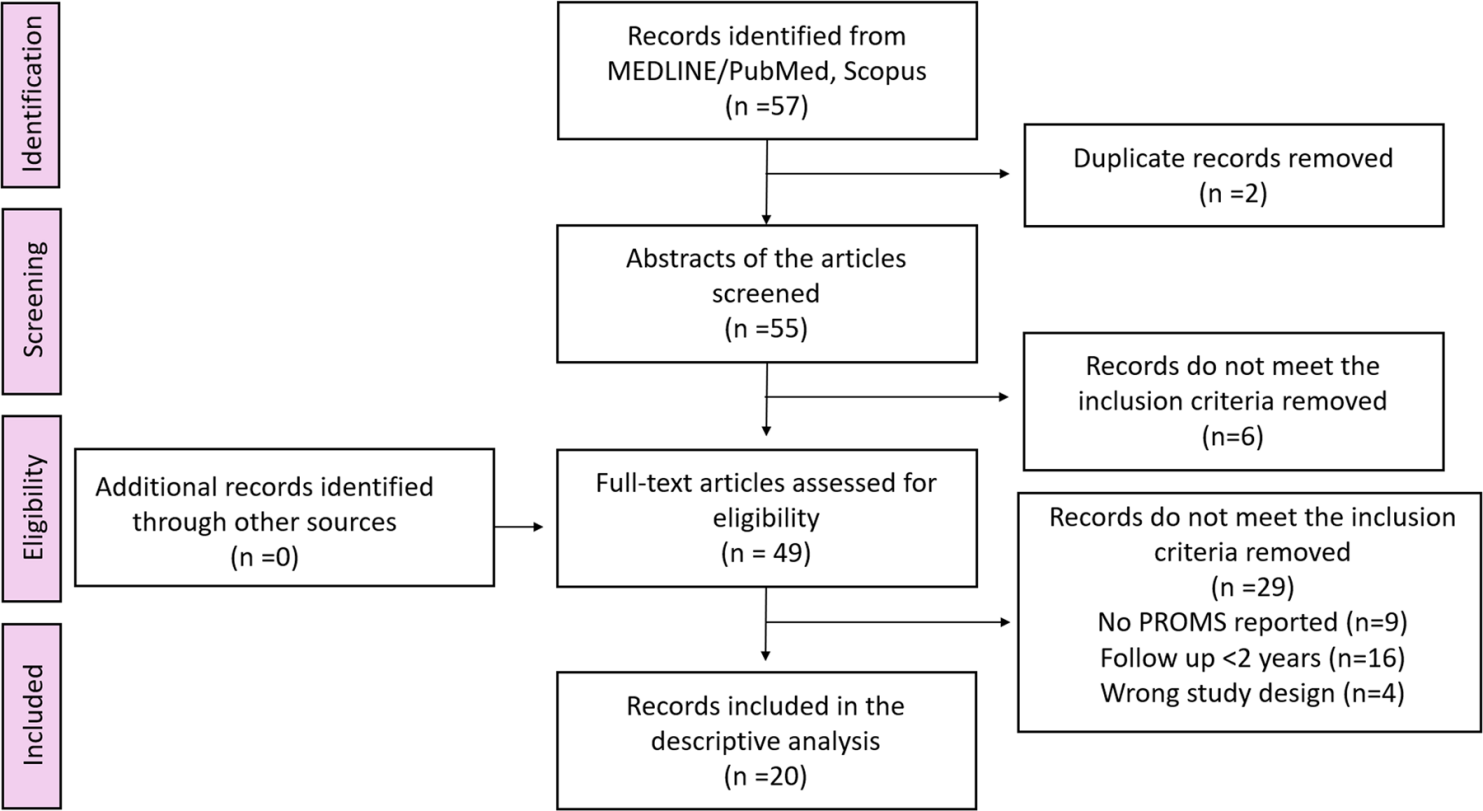
Preferred Reporting Items for Systematic Reviews and Meta‐analyses (PRISMA) flowchart.

The initial database search yielded a total of 57 records. After removal of duplicates and screening of titles and abstracts, 49 studies underwent full‐text review (Figure 1). Twenty studies met the inclusion criteria and were included in the final synthesis. All studies were published between 2023 and 2025.

This review encompassed 5155 primary robotic total knee arthroplasties performed using functional alignment principles [39]. Median cohort size was 292 knees (range, 60–527), with a weighted mean follow‐up of 2.5 years (range, 2–3 years), Most cohorts were retrospective analyses of prospectively maintained registries, while also one randomised trial [44] and one prospective study [8] were included. Most series originated from high‐volume single centres with the Croix Rousse hospital of Lyon contributing several large data sets, and additional studies from South Korea [6], Australia [8], China [45], Italy [10] and New Zealand [44]. Patient demographics were broadly consistent, with mean age between 66 and 72 years, body mass index ranging from 26.3 to 30.4 kg/m², and a female proportion between 58% and 67%. All of the 20 cohorts used the image‐based MAKO (Stryker, Mako Surgical Corp., Fort Lauderdale, FL, USA) platform. Where implant constraint was reported, construct choice was typically mixed but skewed toward posterior‐stabilised (PS) designs (≈ two‐thirds PS) (Table 1).

**Table 1:** Demographic data and implant characteristics of the included studies.

Study	Sample	Age (mean ± SD)	Female (%)	BMI (mean ± SD)	Implant constraint (CR/CS/PS, %)	Fixation type (%)	Follow‐up (years)
Andriollo et al. [3]	310	69 ± 8.3	58.4	28.6 ± 4.9	‐/34.5/65.5	NR	2.9
Andriollo et al. [1]	310	69 ± 8.3	58.4	28.6 ± 4.9	‐/34.5/65.5	NR	2.9
Andriollo et al. [2]	180	69.1 ± 8.3	55.6	28.9 ± 4.6	‐/23.9/76.1	NR	2.9
Choi et al. [6]	60	70.0 ± 5.7	0.78	26.7 ± 2.6	‐/100/‐	NR	2
Clark et al. [8]	300	66−67	49.6−56.9	32.0 ± 8	100	NR	2
Daffara et al. [10]	164	71.7 ± 8.9	62.8	28.7 ± 4.7	51/‐/49	CM (49)	2
						CL (51)	
Diquattro et al. [11]	283	68.6 ± 8.1	55	28.7 ± 4.8	NR/34.3/NR	NR	2.8
Diquattro et al. [12]	268	70 (median)	63.8	28.1 ± 6 (median)	‐/35/65	NR	2.7
Koutserimpas et al. [20]	353	70 (median)	61.8	28.1 (median)	35/‐/65	CM (96)	2.8
						CL (257)	
Koutserimpas et al. [23]	355	70 (IQR 64–74)	57.8	27.8 (IQR 24.9–31.8)	‐/35/65	NR	2.8
Koutserimpas et al. [25]	327	68–70 (median)	58.4	28.1 (IQR 25–32)	35/‐/65	≤3°: CL (70.7) >3°: CL (76.3)	2.5
Koutserimpas et al. [27]	372	70 (IQR 64–74)	59.1	27.8 (IQR 24.9–31.7)	35/‐/65	CM (28) CL (72)	2.5
Koutserimpas et al. [28]	329	71 (IQR 65–75)	66.3	28.1 (IQR 25–32)	‐/37/63	NR	3
Koutserimpas et al. [29]	393	70 (IQR 64–74)	61.3	28.3 (IQR 25.2–31.6)	CM: 3.5/‐/96.5 CL: 42/‐/58	CM (23.5) CL (76.5)	2.5
Koutserimpas et al. [30]	190	70 (IQR 63–75)	54.2	28.3 (IQR 25.5–31.7)	<10°: ‐/73/27 ≥10°: ‐/86/14	NR	3
Manara et al. [34]	101	64.5	35.6	30 (22–40)	CR/CS/‐	CM (tibia & patella) CL (femur)	2
Nixon et al. [37]	527	67.4–67.7	49.2 − 57.2	31.5–32.6	100/‐/‐	NR	2
Yang et al. [43]	100	69.6 ± 6.3	0.8	26.3 ± 4.1	100/‐/‐	CM (100)	2
Young et al. [44]	244	66 ± 7.1	48.8	31.1 ± 4.6	100/‐/‐	CM (100)	2
Yu et al. [45]	110	67 ± 8.1	0.5	26.9 ± 3.7	100/‐/‐	CM (100)	2

Abbreviations: BMI, body mass index; CL, cementless; CM, cemented; CR, cruciate‐retaining; CS, cruciate‐substituting; FA, functional alignment; IQR, interquartile range; PS, posterior stabilise; NR, not reported.

Functional outcomes were reported across all included studies, with the KSS and KSS‐function being the most commonly used measures. The studies demonstrated high postoperative knee function, with the mean KSS Knee score ranging from 92.4 to 94.5, and the mean KSS‐function score ranging from 91.4 to 93.4 (Table 2). The FJS ranged between 74.8 and 77.5 in most studies, with FA principles showing significant differences when compared with conventional mechanical alignment in terms of FJS scores [44]. The Kujala Anterior Knee Pain Scale (AKPS) was another key outcome measure, showing consistent values ranging from 88.1 to 93.2, indicating minimal anterior knee pain across cohorts. Some studies also reported on WOMAC [6] and KOOS Total [8, 10, 37, 43, 44] and OKS [8, 34, 37, 44], indicating an overall high level of knee‐related quality of life post‐surgery. Range of motion (ROM) also varied across studies, with the mean recurvatum being approximately 0.6°–1.1° preoperatively, improving to 0.2°–1.3° postoperatively. The flexion contracture ranged from 2.2° preoperatively to 0.2° postoperatively, with maximum flexion improving from 119.3° preoperatively to a postoperative range of 123°–124.8° (Tables 2 and 3).

**Table 2:** Outcomes score of the included studies.

Study	KSS knee	KSS function	FJS12	AKPS	WOMAC	KOOS total	OKS
Andriollo et al. [3]	92.8–92.9 ± 8	91.4–92.2 ± 9	74.8–77.5 ± 22	88.1–89.4 ± 14	—	—	—
Andriollo et al. [1]	92.4–93.4 ± 8	91.8–92.5 ± 9	CF ≤ 7.5° (76.2 ± 22.1)	88.7–89.9 ± 13	—	—	—
			CF > 7.5° (76.8 ± 21.9)				
Andriollo et al. [2]	92.7 ± 8.7	91.7 ± 10.3	74.8 ± 22.4	88.4 ± 14.1	—	—	—
Choi et al. [6]	94.5 ± 6.4	76.9 ± 15.1	79.6 ± 30.1	—	13.6 ± 12.3	—	—
Clark et al. [8]	—	—	FAk (79.5)	—	—	FAk (86)	FAk (42.7)
			FAm (71.6)			FAm (85.9)	FAm (42.4)
Daffara et al. [10]	—	—	PS (89.3 ± 9.2)	—	—	PS (88.8 ± 10.0)	—
			CR (87.5 ± 12.8)			CR (86.7 ± 14.0)	
Diquattro et al. [11]	93.6 ± 8.4	92.3 ± 8.7	75.0 ± 22.7	89.7 ± 12.0	—	—	—
Diquattro et al. [12]	R (91.7 ± 8.1)	R (90.6 ± 9.9)	R (76.0 ± 23.0)	R (89.5 ± 12.5)	—	—	—
	NR (93.2 ± 8.9)	NR (93.1 ± 8.8)	NR (75.6 ± 22.2)	NR (87.5 ± 15.8)			
Koutserimpas et al. [20]	Age ≥75 (95)	Age ≥75 (94)	—	—	—	—	—
	Age <75 (94.5)	Age <75 (90)					
	Female (94)	Female (90)					
	Male (95)	Male (95)					
Koutserimpas et al. [23]	Varus (95)	Varus (90)	Varus (86)	Varus (92)	—	—	—
	Valgus (94)	Valgus (94)	Valgus (90)	Valgus (95)			
Koutserimpas et al. [25]	≤3° (92.2 ± 8.9)	≤3° (91.3 ± 9.9)	≤3° (75.6 ± 23.9)	—	—	—	—
	>3° (92.6 ± 8.3)	>3° (92.3 ± 8.9)	>3° (77.1 ± 20.9)				
Koutserimpas et al. [27]	BMI ≥ 30 (95)	BMI ≥ 30 (90)	BMI ≥ 30 (86)	—	—	—	—
	BMI < 30 (94.5)	BMI < 30 (90)	BMI < 30 (84)				
Koutserimpas et al. [28]	CS (91)	CS (90)	CS (86)	—	—	—	—
	PS (94)	PS (90)	PS (84)				
Koutserimpas et al. [29]	CL (94)	CL (90)	CL (84)	—	—	—	—
	CM: (95)	CM (90)	CM (90)				
Koutserimpas et al. [30]	≥10° (91)	≥10° (90)	≥10° (80)	≥10° (92)	—	—	—
	<10° (95)	<10° (93)	<10° (82)	<10° (93)			
Manara et al. [34]	—	—	78.4	—	—	—	44 ± 0.8
Nixon et al. [37]	—	—	<2 mm (73.9 ± 2)	—	—	<2 mm (84.4 ± 14.2)	<2 mm (42.0 ± 6.4)
			2–3 mm (72.2 ± 26.6)			2—3 mm (84.8 ± 14.3)	2‐3 mm (42.2 ± 6.8)
			3–6 mm (79.6 ± 23.6)			3‐6 mm (88.4 ± 12.5)	3‐6 mm (43.6 ± 4.9)
Yang et al. [43]	—	—	81.8 ± 7.5	—	—	Pain (84.5)	—
						Symptoms (85.1)	
						ADL (79.1)	
						Sport (25.9)	
						QOL 68.4)	
Young et al. [44]	90.3 ± 10.4	82.6 ± 16.5	70.1 ± 25.6	—	—	86.6 ± 12.9	41.9 ± 5.8
Yu et al. [45]	93.9 ± 6.7	84.6 ± 13.3	—	—	—	—	—

*Note*: Data are expressed as mean ± SD. All KOOS subscales improved.

Abbreviations: AKPS, Kujala Anterior Knee Pain Scale; BMI, body mass index; CF, combined flexion; CL, cementless; CM, cemented; CR, cruciate‐retaining; CS, cruciate‐substituting; EQ‐5D, EuroQol 5 Dimensions; FA, functional alignment; FAk, functional alignment with a preoperative kinematic alignment plan; FAm, functional alignment with a preoperative mechanical alignment plan; FPS, Feller′s patellar score; FJS12, Forgotten Joint Score; FPS, Feller′s patellar score; KOOS, Knee injury and Osteoarthritis Outcome Score; KSS, Knee Society Score; NR, not resurfaced; OKS, Oxford Knee Score; PS, posterior stabilise; R, resurface; SF‐12, 12 items short form survey; WOMAC, Western Ontario and McMaster Universities Arthritis Index.

**Table 3:** Visual analogue score, stair ability and range of motion of the included studies.

Study	VAS	Stair ability	ROM‐r (degrees)	ROM‐c (degrees)	ROM‐f (degrees)
Andriollo et al. [3]	—	—	1.1° ± 2.4–2.5	0.2° ± 1.1–1.3	123.2°–124.8° ± 10
Andriollo et al. [1]	—	—	0.9°–1.5° ± 2−3	0.2°–0.3° ± 1	123.5°–126.4° ± 9–10
Andriollo et al. [2]	—	—	—	—	—
Choi et al. [6]	—	—	—	—	—
Clark et al. [8]	FAk (11.0) FAm (12.3)	—	—	—	FAk (124.6°) FAm (121°)
Daffara et al. [10]	—	—	—	—	—
Diquattro et al. [11]	—	—	1°–1.4° ± 2.3	0.1°–0.3° ± 1.2	124° ± 10.0
Diquattro et al. [12]	—	—	—	—	—
Koutserimpas et al. [20]	—	—	—	—	—
Koutserimpas et al. [23]	—	—	—	—	Varus (130°) Valgus (130°)
Koutserimpas et al. [25]	—	—	0 ± 0	0 ± 0	≤3° (130°) >3° (130°)
Koutserimpas et al. [27]	—	—	BMI ≥ 30 (0) BMI < 30 (0)	BMI ≥ 30 (0) BMI < 30 (0)	BMI ≥ 30 (130°) BMI < 30 (130°)
Koutserimpas et al. [28]	—	—	CS (0) PS (0)	CS (0) PS (0)	CS (130°) PS (130°)
Koutserimpas et al. [29]	—	—	—	—	—
Koutserimpas et al. [30]	—	—	—	≥10° (1°) <10° (0)	≥10° (120°) <10° (130°)
Manara et al. [34]	TKA (9.5 ± 3.5)	—	—	—	TKA (126° ± 2.7)
Nixon et al. [37]	2 mm (10.9 ± 17.9) 2‐3 mm (13.1 ± 21.5) 3‐6 mm (10.1 ± 20.6)	<2 mm (54%)^#^ 2–3 mm (56.7%)^#^ 3–6 mm (70.9%)^#^	—	—	<2 mm (124.2°) 2–3 mm (125°) 3–6 mm (126.3°)
Yang et al. [43]	—	—	—	—	130.1° ± 9
Young et al. [44]	VAS rest 7.6 ± 14.7 VAS mob 9 ± 15.9	—	0 ± 0	0 ± 0	120.2° ± 9.4
Yu et al. [45]	0.7 ± 0.9	—	—	—	113.4° ± 8

*Note*: Data are expressed as mean ± SD.

Abbreviations: BMI, body mass index; CS, cruciate‐substituting; FA, functional alignment; FAk, functional alignment with a preoperative kinematic alignment plan; FAm, functional alignment with a preoperative mechanical alignment plan; PS, posterior stabilise; ROMf, range of motion‐flexion; ROM‐r, range of motion‐recurvatum; ROMc, range of motion‐contracture; TKA, total knee arthroplasty; UKA, unicompartmental knee arthroplasty; VAS, visual analogue score.

Complication and early revision rates were low throughout the included series. The most frequent events were stiffness requiring manipulation under anaesthesia (*N* = 92; 1.78%), and haematomas (*N* = 63; 1.22%). Mechanical failures were observed more frequently (4 cases – 3%) in obese patients, as described by Koutserimpas et al. [27], while rare revisions for aseptic loosening, instability, or allergic reactions were also reported. Among these cohorts, 18 early aseptic revisions were reported, with eight cohorts having no events [3, 6, 11, 12, 34, 43, 44, 45] and the rest 12 cohorts reporting at least one revision. The overall pooled proportion of early aseptic revisions was 0.35% (18 revisions out of 5155 knees; 95% CI: 0.23%–0.55%) (Table 4).

**Table 4:** Complications of the included studies.

Study	Complication, *N* (%)	Infection	MUA	Other complications (*N*)	Early aseptic revisions, *N* (%)	Revision indications
Andriollo et al. [3]	21 (6.8)	2	13	Scar revision (3)	2 (0.6)	Nickel allergy
				Nickel allergy revision (1)		Femoral revision
				Patellar revision (1)		Patellar revision
				Femoral revision (1)		
Andriollo et al. [1]	21 (6.8)	2	13	Scar revision (3)	2 (0.6)	Nickel allergy
				Nickel allergy revision (1)		Femoral revision
				Patellar revision (1)		Patellar revision
				Femoral revision (1)		
Andriollo et al. [2]	0 (0)	0	0	NR	0 (0)	NR
Choi et al. [6]	1 (1.7)	0	0	Femoral shaft pin‐site fracture	0 (0)	NR
Clark et al. [8]	1 (0.3)	1	4	Fracture (1)	1 (0.3)	Pain (1)
						Infection (1)
Daffara et al. [10]	3 (1.8)	2	NR	NR	1 (0.6)	PS: instability (1), infection (1)
						CR: infection (1)
Diquattro et al. [11]	0 (0)	0	NR	NR	0 (0)	0
Diquattro et al. [12]	0 (0)	0	0	NR	0 (0)	NR
Koutserimpas et al. [20]	46 (13)	3	18	Haematoma (16)	1 (0.3)	Mechanical failure (aseptic)
				Wound (3)		
				Intra‐operative femoral fracture (3)		
				Reoperation patellar resurfacing (2)		
Koutserimpas et al. [23]	4 (1.1)	1	NR	NR	1 (0.3)	Varus: Aseptic loosening (1)
Koutserimpas et al. [25]	Total: 37 (11.3);	≤3°: DAIR (1)	Total: 14	≤3°: haematoma (5), DVT (2), delayed wound healing (2)	1 (0.3)	>3°: aseptic (1)
	≤3°:19 (12.7)	>3°: DAIR (1)	≤3°: (9)	>3°: haematoma (9), delayed wound healing (1), patella button removal (1)		
	>3°: 18 (10.2)		>3°: (5)			
Koutserimpas et al. [27]	Total: 47 (12.6);	NR	NR	BMI ≥ 30: septic & aseptic (4.5%)	3 (0.8)	BMI ≥ 30: mechanical failures (4)
	BMI ≥ 30: 30 (12.7)			BMI < 30: aseptic (3%)		BMI < 30: mechanical failures (1)
	BMI < 30: 17 (13.5)					
Koutserimpas et al. [28]	Total: 40 (12.2)	PS: 3	Total: 15	CS: haematomas (5)	3 (0.9)	CS: femoral instability (2)
	CS: 13 (10.7)		CS: 6	PS: lateral condyle fractures (3)		PS: aseptic (1)
	PS: 27 (13)		PS: 9	haematomas (11)		
Koutserimpas et al. [29]	Total: 41	Total: 5	Total: 14	CL: haematomas (17), femoral fractures (1)	1 (0.3)	CL: aseptic (1)
	CL: 34 (12.3)	CL: 3	CL: 12	CM: femoral fractures (2), medial condyle necrosis (1)		CM: fracture (1)
	CM: 7 (8.2)	CM: 2	CM: 2			
Koutserimpas et al. [30]	≥10°: 1 (2.3)	≥10°: 1	NR	<10°: femoral component (1)	1 (0.5)	≥10°: DAIR (1), femoral revision (1)
	<10°: 2 (1.4)	<10°: 1				<10°: DAIR (1)
Manara et al. [34]	NR	NR	NR	TKA: cellulitis (1)	0	NR
Nixon et al. [37]	6 (1.1)	2	NR	Instability (2)	2 (0.4)	2–3 mm: instability (2)
Yang et al. [43]	0 (0)	0	0	0	0 (0)	0
Young et al. [44]	2 (0.8)	1	1	NR	0 (0)	1 infection
Yu et al. [45]	0 (0)	0	0	NR	0 (0)	NR

Abbreviations: BMI, body mass index; CL, cementless; CM, cemented; CR, cruciate‐retaining; CS, cruciate‐substituting; DAIR, debridement antibiotics implant retention; DVT, deep venous thrombosis; FA, functional alignment; MUA, manipulationa under anaesthesia; NR, not reported; PS, posterior stabilise.

**Figure 2:**
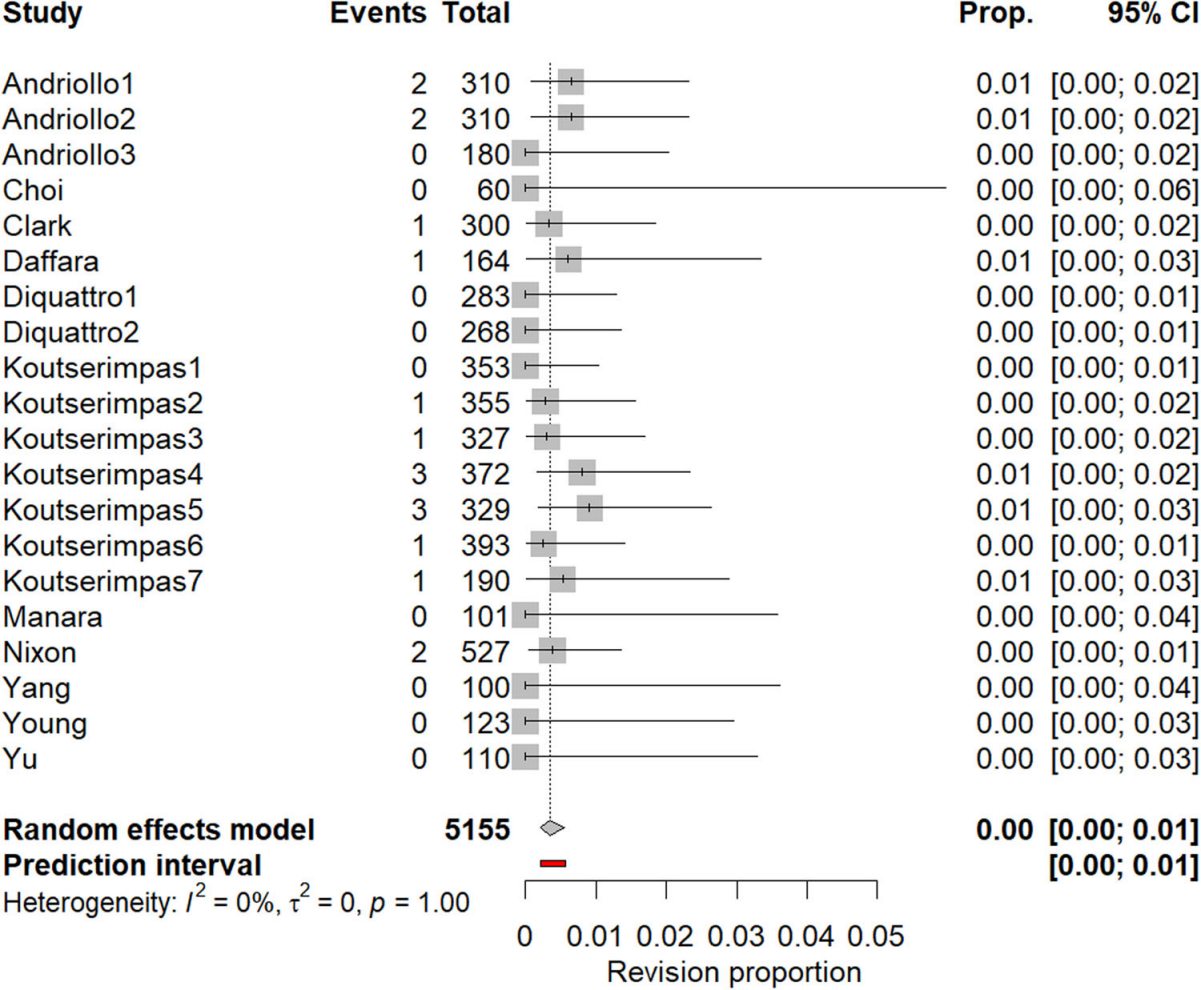
Forest plot of early aseptic revision proportions after functionally aligned robotic total knee arthroplasty. Andriollo (1), (2), (3) refers to [1, 2, 3], Diquattro (1) and (2) refer to studies [10] and [11], Koutserimpas (1), (2), (3), (4), (5), (6), (7) refers to studies [23, 25, 27, 28, 29, 30] respectively. CI, confidence interval.

**Figure 3:**
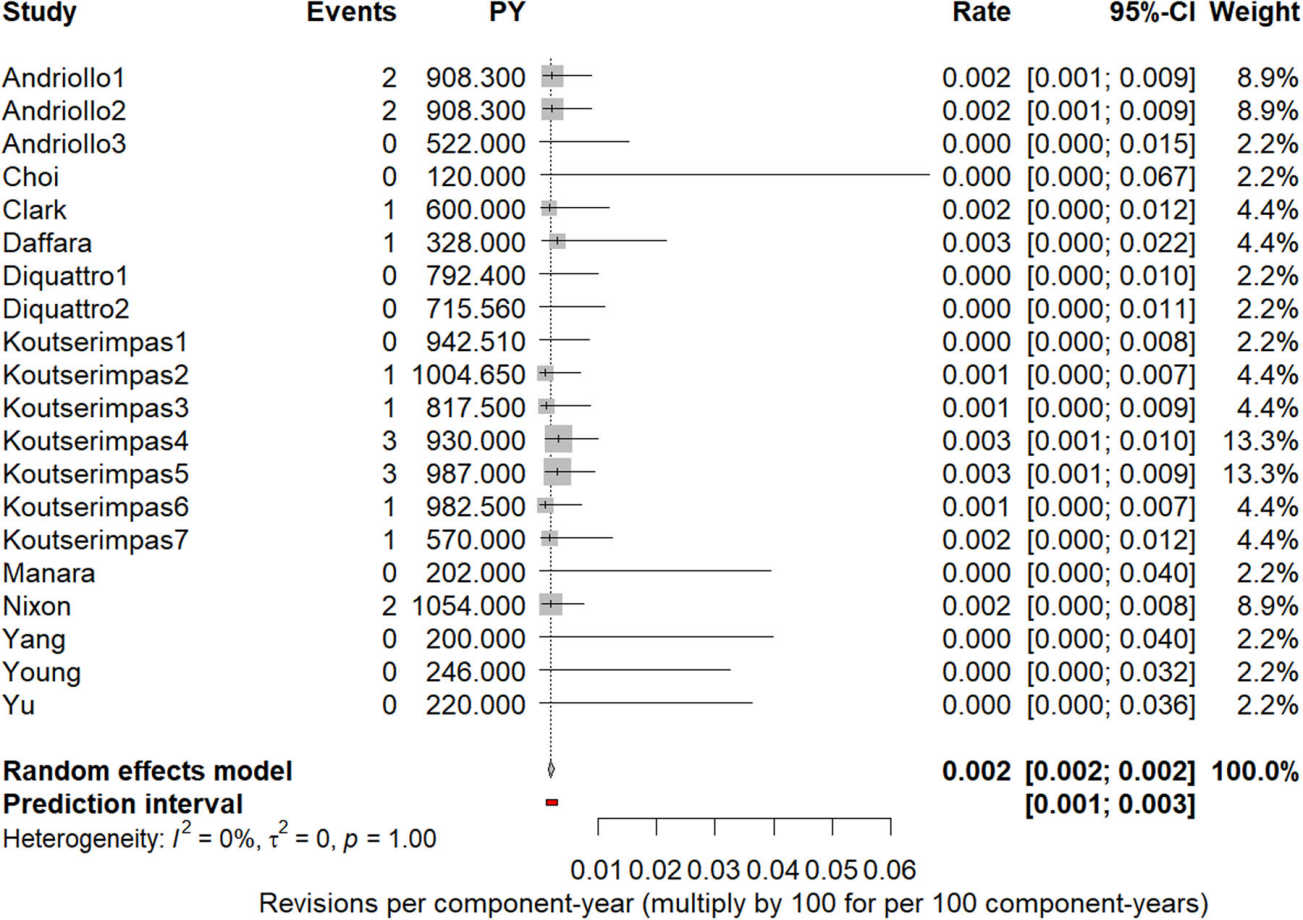
Forest plot of early aseptic revision incidence rates (revisions per component‐year). Andriollo (1), (2), (3) refers to [1, 2, 3], Diquattro (1) and (2) refer to studies [10] and [11], Koutserimpas (1), (2), (3), (4), (5), (6), (7) refers to studies [23, 25, 27, 28, 29, 30] respectively.CI, confidence interval.

**Figure 4:**
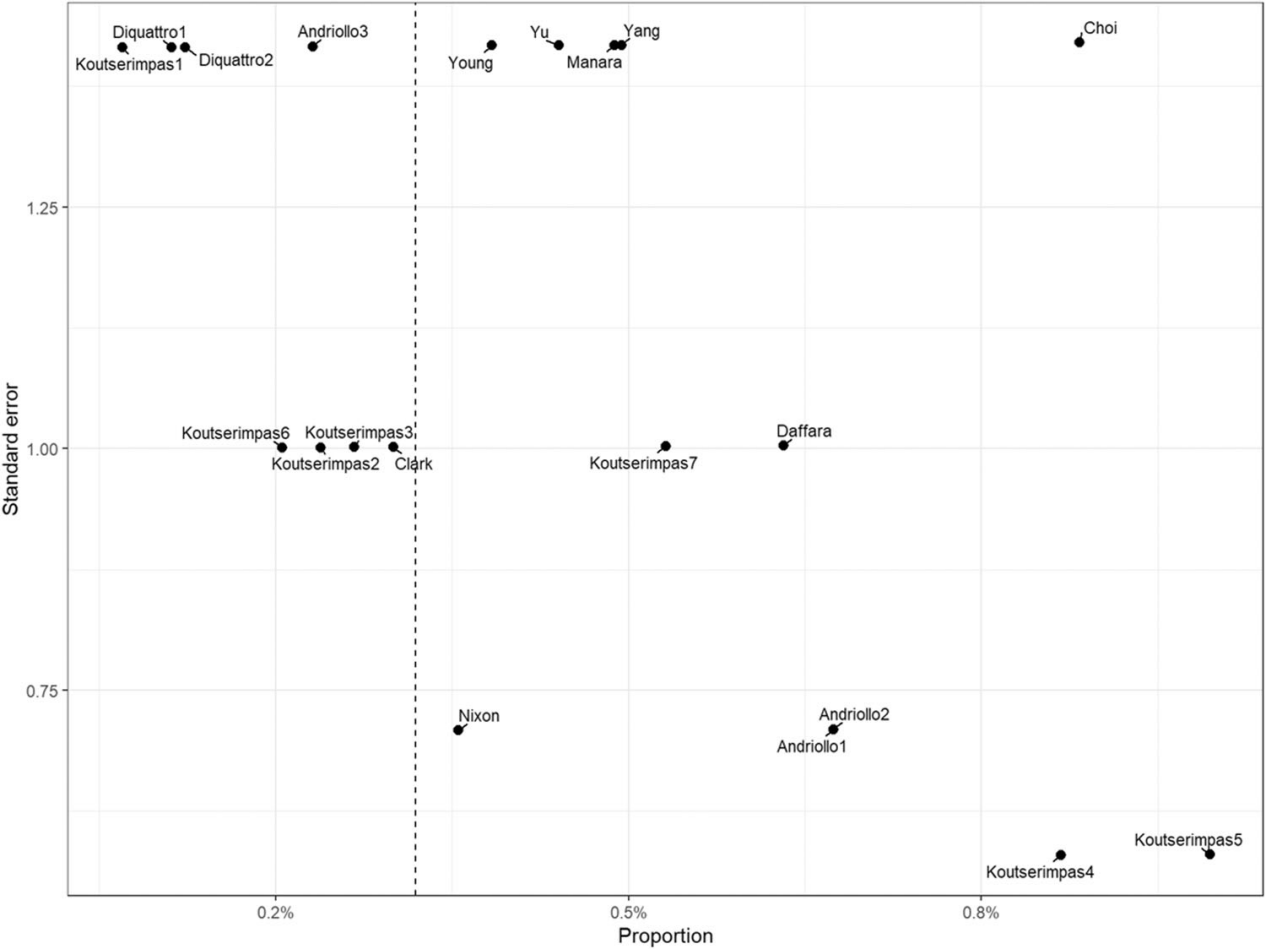
Funnel plot of early aseptic revision proportions. Andriollo (1), (2), (3) refers to [1, 2, 3], Diquattro (1) and (2) refer to studies [10] and [11], Koutserimpas (1), (2), (3), (4), (5), (6), (7) refers to studies [23, 25, 27, 28, 29, 30] respectively.

The single‐arm meta‐analysis of these cohorts demonstrated a very low revision risk with negligible variability across studies. The random‐effects pooled proportion of early aseptic revisions was effectively zero, rounded to 0.00 (95% CI: 0.00–0.01), with *I*² ≈ 0% and *p* = 1.00 (Figure 2). The pooled incidence rate of revisions per component‐year was also very low, approximately 0.2 per 100 component‐years, with *I*² ≈ 0% and *p* = 1.00 (Figure 3). The funnel plot showed no significant asymmetry, indicating no evident small‐study effects, although the limited number of events and narrow study size range restricts statistical power to detect such effects (Figure 4).

### Study quality (ROBINS‐I)

Risk‐of‐bias assessments for the included observational cohorts were primarily moderate (18 cohorts), with a small number of studies rated as low‐moderate (2 cohorts). The sole randomised controlled trial was assessed as having a low risk of bias based on the ROB2 tool used for randomised studies. Formal small‐study effect testing showed no evidence of bias: Peters' test for the proportion meta‐analysis non‐significant (*p* > 0.10) and Egger's regression for the incidence‐rate meta‐analysis non‐significant (*p* > 0.10). Power is limited due to the very low event rates and the narrow study‐size range.

## DISCUSSION

The most important finding of this study was that robotic FA TKA is associated with favourable patient‐reported outcomes, low complication rates, and very low rates of aseptic revision across more than 5000 patients at 2.5 years of follow‐up.

These results sit within the broader debate on alignment strategies. Mechanical alignment is reproducible but it may disregard patient‐specific morphology [4, 5, 9, 33]. Kinematic alignment (KA), while attractive for restoring native joint lines and often favourable patellofemoral kinematics, may be problematic in valgus knees: robotic simulations and technical reports have shown that KA may drive excessive femoral valgus and internal rotation in valgus morphotypes; changes that risk undercoverage or malorientation of the trochlea and potentially aggravate anterior compartment mechanics unless implant design is specifically adjusted [19, 40, 41]. These concerns have prompted proposals for explicit “boundaries” when applying KA, particularly in valgus alignment. By contrast, FA, implemented with robotics, has been applied successfully in valgus knees, achieving safe coronal alignment within defined targets, avoiding soft‐tissue releases, and delivering comparable outcomes and survivorship to varus cases, without adverse signals related to component positioning or the anterior compartment [17, 23]. While early evidence suggests advantages of FA in restoring joint line and ligament balance, comparative data against mechanical and KA remain limited. Direct comparative studies will be needed to define whether these theoretical benefits translate into meaningful clinical differences.

FA, or functional knee positioning, follows a reproducible intraoperative workflow that begins with robotic planning to balance the medial and lateral gaps in extension and flexion while preserving joint line height and respecting safe boundaries of coronal, sagittal, and rotational component positioning [16, 22, 39]. Notably, fourteen cohorts explicitly followed the Shatrov et al study for FA, targeting HKA 174°–180° and component positioning within 3° varus to 6° valgus for the femur and 0°–6° varus for the tibia [39]. The remaining studies used comparable limits (±3° to ±5°), indicating that FA was applied within a narrow and reproducible range across the included literature. Within this framework, a recurrent concern has been the management of marked varus deformities, particularly regarding tibial implant positioning. Two main strategies have been described: expanding the tibial varus beyond the conventional 3° threshold (up to ~6°) to restore native alignment without soft tissue release, or alternatively respecting the ≤3° varus limit and performing limited medial releases to achieve balance [18, 25]. Both approaches have been applied successfully, with recent series reporting excellent functional outcomes and negligible differences in complications or implant survivorship. Importantly, this meta‐analysis confirmed that even in cohorts where tibial varus positioning exceeded 3°, the rate of aseptic revision remained minimal, reinforcing the safety of FA strategy [18, 25].

Beyond the coronal plane, FA also addresses the sagittal and axial planes, with particular emphasis on the anterior compartment [1, 2]. This “third space” has historically received limited attention in mechanically aligned TKA, where femoral component rotation was used as a surrogate for patellofemoral mechanics [4]. In contrast, FA explicitly and independently evaluates the anterior compartment by matching the native trochlea to the anterior flange of the femoral component, as well as dynamically visualising the patellofemoral tracking [21, 31]. This approach aims to optimise trochlear tracking in the mediolateral aspect but also to avoid overstuffing or understuffing, and respect the native patellofemoral morphology, taking into account that overstuffing of the anterior compartment >5 mm leads to adverse outcomes [26]. Recent studies have confirmed that FA with the use of the image‐based robotic system achieves accurate restoration of patellar tilt, translation, and offset, reducing reliance on patellar resurfacing and minimising the risk of anterior knee pain [11]. Importantly, both resurfaced and non‐resurfaced cases have shown excellent outcomes when the anterior compartment is restored using FA, indicating that robotic assessment and dynamic visualisation of the patellofemoral joint can reliably safeguard implant positioning and extensor mechanism function [12, 15].

Another important aspect is whether patient‐ or implant‐related variables influence outcomes under FA principles. A recent study demonstrated that FA yields consistent outcomes across gender and age groups, with elderly patients (≥75 years) and women achieving comparable functional scores, complication rates, and survivorship to younger or male patients [20]. In contrast, BMI appears to play a more critical role. While functional results remain excellent across all weight categories, obese patients have shown a higher incidence of mechanical complications and aseptic revisions, indicating that excess body weight may still predispose to implant‐related failure despite the precision of FA [27]. Implant choice also appears flexible under FA: both cruciate‐substituting and PS inserts have shown equivalent results in robotic TKA, with no differences in functional scores or revision rates [28]. Fixation method is another debated variable, but comparative analyses confirm that both cemented and cementless implants are safe in FA, with similar mid‐term survivorship and functional outcomes, despite minor differences in radiographic tibial varus positioning [29]. Finally, FA has been shown to manage preoperative flexion contracture ≥10° without requiring soft‐tissue releases, achieving functional outcomes and survivorship equivalent to patients without contracture [30]. Taken together, these findings reinforce that FA provides stable and reproducible results across diverse demographic profiles and implant designs, while highlighting that BMI remains a potential risk factor for mechanical complications and should be considered carefully in surgical planning.

Robotic‐assisted TKA platforms that provide quantitative, real‐time gap measurements and soft‐tissue balance information appear to offer clear advantages; particularly when applying FA. For example, Clark et al. showed that FA achieved “overall balance” in ~97% of knees (within ±2 mm in extension/flexion/medial‐lateral) compared to 73% with kinematic and 55% with mechanical alignment, before any soft‐tissue releases [7]. Similarly, Lee et al. demonstrated that FA required significantly less additional soft tissue release compared to mechanical alignment, performed with the manual or robotic technique, with better patient‐reported outcomes at 1 year [32]. These data suggest that the capabilities of robotic systems to assess gaps, visualise balance, and fine‐tune component position make FA more effective in achieving soft‐tissue sparing, more physiological alignment, and better early outcomes than alignment strategies that do not leverage these intraoperative measurements. Nevertheless, it should be noted that the majority of the included studies did not quantify the force applied during intraoperative gap assessment. Because manual tensioning varies between surgeons, this may influence gap measurements and implant positioning. Lack of objective force measurement remains a limitation of the current evidence.

A previous review by Migliorini et al. summarised early clinical experience with FA, reporting promising short‐term outcomes in 1198 patients (mean follow‐up ~17 months) [35]. The present review builds on those findings by analysing over 5000 patients and including only studies with a minimum 2‐year follow‐up, offering a more contemporary appraisal. In addition, we also reported pooled aseptic revision rates, which were not analysed in the previous review.

This review has some limitations that should be considered. Most of the included cohorts were retrospective single‐center series, with only one randomised controlled trial available, which may introduce selection and reporting bias. The follow‐up was limited to 2–3 years; however, this is inherent to the fact that FA is a relatively new concept. In addition, some studies did not report all complication categories, which may lead to underestimation of overall event rates. Moreover, the *I*² value of 0% could reflect the low number of revision events and small effect size, and therefore may underestimate true heterogeneity. Another limitation is the heterogeneity in outcome reporting, as studies used different PROMs (KSS, FJS, WOMAC, OKS, Kujala and KOOS), which limited pooled analyses across all functional domains. Also, most studies did not evaluate outcomes according to patient phenotypes (such as severe varus or valgus, obesity, or flexion contracture), and future research focusing on phenotype‐specific results could provide valuable information for refining FA strategies further. Finally, it should be noted that a significant proportion of included patients originated from a single high‐volume center, which may limit generalisability. In addition, some Lyon‐based cohorts likely include overlapping patients, as several studies reported results from institutional series with similar timeframes.

## CONCLUSION

This systematic review has demonstrated that robotic‐assisted functionally aligned TKA demonstrates favourable PROMS and very low rates of complications and aseptic revision at 2.5 years of follow‐up. While longer‐term and multicenter data are still needed, the available evidence suggests that FA is a safe and reproducible alignment strategy in contemporary TKA.

## AUTHOR CONTRIBUTIONS

*Conceptualisation*: Vasileios Giovanoulis, Angelo V. Vasiliadis, Luca Andriollo, Pietro Gregori, Christos Koutserimpas, Konstantinos Dretakis, and Sébastien Lustig. *Data curation*: Luca Andriollo, Pietro Gregori, Konstantinos Dretakis, Vasileios Giovanoulis, and Angelo V. Vasiliadis. *Formal analysis*: Vasileios Giovanoulis, Angelo V. Vasiliadis, and Christos Koutserimpas. *Investigation*: Luca Andriollo, Pietro Gregori, Konstantinos Dretakis, Christos Koutserimpas, and Sébastien Lustig. *Methodology*: Konstantinos Dretakis, Christos Koutserimpas, and Sébastien Lustig. *Project administration*: Konstantinos Dretakis and Sébastien Lustig. *Supervision*: Konstantinos Dretakis, Christos Koutserimpas, and Sébastien Lustig. *Validation*: Pietro Gregori, Luca Andriollo, and Christos Koutserimpas. *Visualisation*: Vasileios Giovanoulis and Angelo V. Vasiliadis. *Writing–original draft*: Vasileios Giovanoulis, Angelo V. Vasiliadis, Luca Andriollo, Pietro Gregori, and Christos Koutserimpas. *Writing–review and editing*: Konstantinos Dretakis and Sébastien Lustig.

## CONFLICT OF INTEREST STATEMENT

Konstantinos Dretakis: Consultant for Stryker. Christos Koutserimpas: Consultant for Smith and Nephew. Sébastien Lustig: Consultant for Stryker. The remaining authors declare no conflicts of interest.

## ETHICS STATEMENT

The protocol of this review has been prospectively registered with the International Prospective Register of Systematic Reviews (PROSPERO) (registration number: CRD420251134340).

## Data Availability

The data that support the findings of this study are available from the corresponding author, upon reasonable request.
